# Pediatric Pleomorphic Adenoma of the Parotid: Case Report, Review of Literature and Novel Therapeutic Targets

**DOI:** 10.3390/children5090127

**Published:** 2018-09-18

**Authors:** Girish Gulab Meshram, Neeraj Kaur, Kanwaljeet Singh Hura

**Affiliations:** 1 Department of Pharmacology, Postgraduate Institute of Medical Education and Research and Dr. Ram Manohar Lohia Hospital, New Delhi 110001, India; 2 Department of Radiology, The University of Texas Health Science Centre, San Antonio, TX 78229, USA; neerajkaur99@gmail.com; 3 Department of Paediatrics, Richmond University Medical Centre, Staten Island, NY 10310, USA; kanwaljeetsinghhura@gmail.com

**Keywords:** pleomorphic adenoma of parotid, salivary gland tumors, parotidectomy

## Abstract

Salivary gland tumors are extremely rare and encompass a diverse group of histologies. Less than 5% of the affected population is pediatric. We present a case of 6-year-old child with pleomorphic adenoma of the parotid. The patient underwent a superficial parotidectomy. Recurrence was not observed in the six months of follow-up. Surgery is the mainstay of the management of benign salivary gland tumors. Although novel molecular agents are being explored, personalized therapy would be a challenge due to the rarity and vast genetic/histologic variations of salivary gland tumors.

## 1. Introduction

Salivary gland carcinomas are exceedingly rare in the pediatric population. Their annual incidence is estimated to be around one case per million [1]. The majority of pediatric tumors of the salivary gland are benign, with pleomorphic adenoma being the most common type [2]. There is a paucity of clinical and biological details about pediatric salivary gland tumors and their clinical behavior in the literature [1,3]. We present a rare case of pleomorphic adenoma of the parotid gland in a 6-year-old child. We also discuss some of the novel molecular targets under evaluation for treating salivary gland malignancies.

## 2. Case Presentation

**Figure 1:**
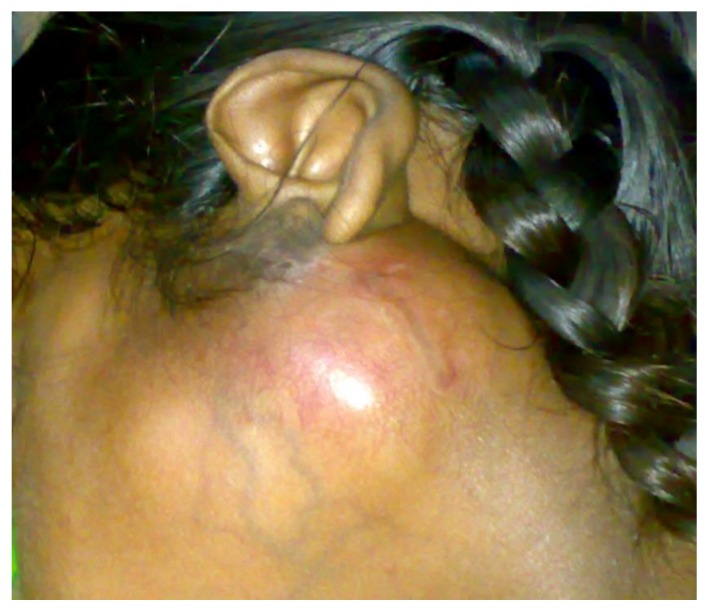
Initial presentation of the patient with a well-defined swelling below the left earlobe.

A 6-year-old female child approached the pediatric outpatient department with a swelling under her left ear that had been present for the past one year. The swelling had gradually increased to its present size and was well-defined, multilobular, 5 cm × 4 cm in diameter, and erythematous. The left ear was slightly everted as shown in Figure 1. On palpation, the swelling was firm, non-tender, and affixed to the surrounding structures. Lymph node palpation and facial nerve palsy was absent. Magnetic resolution imaging (MRI) of the lesion was done. T2-weighted images exhibited a 40 mm × 34 mm sized, well-defined, high-intensity, heterogeneous mass arising from the superficial lobe of the left parotid, which had displaced the surrounding soft tissue. The deep lobe of the parotid and the facial nerve were not involved. Fine needle aspiration cytology (FNAC) was consistent with pleomorphic adenoma. Other routine blood investigations were within normal range. A clinical diagnosis of pleomorphic adenoma of the parotid was made. The patient subsequently underwent superficial parotidectomy with preservation of the facial nerve under general anesthesia. No recurrence has been observed in six months of follow-up.

## 3. Discussion

Salivary gland tumors represent 0.3% of all malignancies and account for around 5% of all head and neck cancers. Less than 5% of the affected population is pediatric [2,4]. Almost 80% of salivary gland tumors arise in the parotid gland followed by the submandibular gland. Approximately 80% of all parotid tumors are benign [4,5]. Common benign parotid tumors include pleomorphic adenoma and pilomatrixoma. The most common malignant parotid tumor is mucoepidermoid carcinoma [6].

Most cases with parotid tumors present with history of a slowly enlarging mass, as found in our patient, or are incidentally discovered by physicians during routine examination [7]. Some reports suggest ultrasound imaging as an initial investigation to differentiate cystic/vascular lesions from solid masses [5]. However, MRI is a more comprehensive diagnostic tool which helps in preoperative assessment of the local-regional extent of the mass, detection of facial nerve and regional lymph node involvement, and differentiation from other malignancies/vascular tumors [1]. Ultrasound-guided FNAC helps in determining the subtype of the parotid tumor on the basis of histopathology. It can be conducted only after vascular tumors are ruled out. Salivary gland tumors (both benign and malignant) have been classified into 24 distinct histological subtypes by the World Health Organization [8]. In our patient, FNAC findings showed the presence of epithelial cells, myoepithelial cells, and mucoid material. Hence, the tumor was classified as pleomorphic adenoma of the parotid.

Superficial parotidectomy with en-bloc excision of the tumor mass, with preservation of the facial nerve, is the surgery of choice for parotid pleomorphic adenoma involving the superficial lobe [3]. If the deeper lobe is involved, then total or radical parotidectomy is the procedure of choice, depending on the extent of the tumor mass and involvement of the facial nerve [4]. Following surgery, recurrence rates are as low as 0–2% [3]. The facial nerve in children is located more superficially and is more sensitive to dissection/stretching than in adults. Hence, facial nerve monitoring during parotidectomy is recommended to reduce the risk of facial nerve palsy/paresis [4]. There is no role of systemic chemotherapy and radiotherapy in benign parotid tumors [8]. As most patients can tolerate pleomorphic adenoma of the parotid for a long duration without any symptoms or major discomfort, they often delay medical advice. However, such delays increase the risk of malignant transformation [9].

As in the case of benign parotid tumors, surgery is also the mainstay in the management of malignant parotid tumors [1,9]. Simultaneous neck dissection is indicated in high-grade malignancies, wherein lymph node involvement is confirmed both clinically and radiologically [1,7]. Adjuvant radiotherapy is considered in cases of incomplete surgical resection, persistent lymph node involvement, perineural invasion, or an aggressive histological grade tumor [4,7]. However, the risk of post-irradiation complications such as facial/dental deformities, secondary malignancies, trismus, and hyposialia should be carefully evaluated [1,4,7]. Perioperative systemic chemotherapy is reserved for malignant forms of salivary gland tumors which are rapidly progressing, recurrent, metastatic, incurable, or unresectable. There are a few trials evaluating the role of systemic monotherapy with cisplatin, vinorelbine, paclitaxel, and eribulin in such cases [9]. Combination regimens evaluated in Phase 2 trials include cyclophosphamide, doxorubicin, cisplatin (CAP regimen) and gemcitabine ± cisplatin [8,9]. However, the risk-benefit ratio must be carefully evaluated before initiating systemic chemotherapy. Due to the indolent course of salivary gland malignancies, several oncologists prefer watchful waiting over systemic chemotherapy [9].

Genomic and molecular profiling of salivary gland tumors has led to a spike in clinical trials evaluating novel molecular targets. Overexpression of c-kit, epidermal growth factor receptor (EGFR), human epidermal growth factor receptor 2 (HER2), vascular endothelial growth factor (VEGF), and androgen receptor (AR) have been observed in various malignant forms of salivary gland tumors [8]. However, Phase 2 clinical trials evaluating imatinib (c-kit inhibitor), geftinib (EGFR inhibitor), cetuximab (monoclonal antibody against EGFR), trastuzumab (monoclonal antibody against HER2) and lapatinib (dual tyrosine kinase inhibitor of EGFR and HER2) did not show very encouraging results. Phase 2 clinical trials on sorafenib, and sunatinib (inhibitors of VEGF) were marked by several adverse effects. Clinical trials on AEE788 (dual inhibitor of VEGF and EGFR), lenvatinib (VEGF inhibitor) and bevacizumab (monoclonal antibody against VEGF) are yet to be completed [8,9]. Molecules inhibiting the fibroblast growth factor receptor (dovitinib), nuclear factor-kappa B pathway (bortezomib), and cytotoxic T-lymphocyte-associated protein 4 (ipilimumab), are being explored in Phase 2 clinical trials. Clinical trials evaluating the role of pazopanib/nintedanib/dasatinib (tyrosine kinase inhibitors), tamoxifen (AR inhibitor), bicalutamide + triptorelin (AR inhibitor + GnRH agonist) and temsirolimus (mammalian target of rapamycin inhibitor) are also underway [10]. Other molecules being evaluated include pembrolizumab (monoclonal antibody against programmed cell death receptor 1), vemurafenib (BRAF inhibitor), nivolumab (monoclonal antibody against BRAF), and AZD451 (TRKC/NTRK3 inhibitor) [8,9,10].

## 4. Conclusions

Salivary gland tumors are an exceedingly rare entity. Superficial parotidectomy is the cornerstone in the management of pleomorphic adenoma of the parotid gland. Malignant tumors of the salivary gland require more invasive surgeries with or without chemotherapy/radiotherapy. New molecular targets and chemotherapeutic agents for malignant forms of salivary gland tumors are being explored in various stages of clinical trials. However, due to their rarity and wide genetic/molecular/histologic profile it would be a challenge to develop a personalized therapeutic approach for salivary gland malignancies.
